# Establishing the relationships between adiposity and reproductive factors: a multivariable Mendelian randomization analysis

**DOI:** 10.1186/s12916-023-03051-x

**Published:** 2023-09-12

**Authors:** Claire Prince, Laura D. Howe, Gemma C. Sharp, Abigail Fraser, Rebecca C. Richmond

**Affiliations:** 1 MRC Integrative Epidemiology Unit, University of Bristol, Bristol, UK; 2 Population Health Sciences, Bristol Medical School, University of Bristol, Bristol, UK; 3 School of Psychology, University of Exeter, Exeter, UK

**Keywords:** Reproductive factors, Adiposity, Mendelian randomization, UK Biobank

## Abstract

**Background:**

Few studies have investigated associations between adiposity and reproductive factors using causal methods, both of which have a number of consequences on women’s health. Here we assess whether adiposity at different points in the lifecourse affects reproductive factors differently and independently, and the plausibility of the impact of reproductive factors on adiposity.

**Methods:**

We used genetic data from UK Biobank (273,238 women) and other consortia (EGG, GIANT, ReproGen and SSGAC) for eight reproductive factors: age at menarche, age at menopause, age at first birth, age at last birth, number of births, being parous, age first had sexual intercourse and lifetime number of sexual partners, and two adiposity traits: childhood and adulthood body size. We applied multivariable Mendelian randomization to account for genetic correlation and to estimate the causal effects of childhood and adulthood adiposity, independently of each other, on reproductive factors. Additionally, we estimated the effects of reproductive factors, independently of other relevant reproductive factors, on adulthood adiposity.

**Results:**

We found a higher childhood body size leads to an earlier age at menarche, and an earlier age at menarche leads to a higher adulthood body size. Furthermore, we find contrasting and independent effects of childhood and adulthood body size on age at first birth (beta 0.22 SD (95% confidence interval: 0.14, 0.31) vs − 2.49 (− 2.93, − 2.06) per 1 SD increase), age at last birth (0.13 (0.06,0.21) vs − 1.86 (− 2.23, − 1.48) per 1 SD increase), age at menopause (0.17 (0.09, 0.25) vs − 0.99 (− 1.39, − 0.59) per 1 SD increase), and likelihood of having children (Odds ratio 0.97 (0.95, 1.00) vs 1.20 (1.06, 1.37) per 1 SD increase).

**Conclusions:**

Our findings demonstrate the importance of considering a lifecourse approach when investigating the inter-relationships between adiposity measures and reproductive events, as well as the use of ‘age specific’ genetic instruments when evaluating lifecourse hypotheses in a Mendelian randomization framework.

**Supplementary Information:**

The online version contains supplementary material available at 10.1186/s12916-023-03051-x.

## Background

Multiple observational studies have shown associations between women’s reproductive factors including age at menarche (AAM) [1, 2], age at first birth (AFB) [1, 3], number of births [1, 4], age at menopause (AMP) [5–7], and measures of adiposity. It has previously been shown that adiposity experienced at different points in the lifecourse has independent effects on later life outcomes, including smoking behaviours, coronary heart disease, type 2 diabetes, and breast cancer [8, 9]. It is also plausible that adiposity experienced in childhood may have different effects on reproductive factors than adiposity or weight gain experienced later in life [10].

There remain a number of areas of active research that will aid in emphasizing the importance of using a lifecourse approach when investigating adiposity and reproductive events, and untangling the interplay between these factors as a basis for understanding mechanisms of disease:

1. Whether adiposity at different points in the lifecourse affects reproductive factors, i.e. adiposity experienced in childhood and adulthood.
2. Whether any effects of adiposity at one point in the lifecourse, e.g. childhood, on reproductive factors is independent of adiposity at other points in the lifecourse, e.g. adulthood.
3. Whether it is also plausible that reproductive factors affect adiposity, e.g. menopausal stage affecting concurrent and post-menopausal adiposity [11].
  Few studies have used causal methods to investigate the associations between adiposity and reproductive factors, particularly considering time-varying adiposity [12]. Mendelian randomization (MR) is a method that can avoid problems of confounding and reverse causality, allowing the assessment of causality by using genetic variants associated with an exposure of interest as instrumental variables [13].

Two MR studies: one using the UK Biobank study and the other the Avon Longitudinal Study of Parents and Children, with replication in the Genetic Investigation of Anthropometric Traits (GIANT) consortium, have suggested that earlier AAM causes higher adulthood body mass index (BMI) [14, 15]. However, the effect identified in the second study attenuated when adjusted for childhood BMI [15]. Others have identified evidence to suggest a strong causal effect of higher childhood BMI on the risk of early menarche (i.e. a potential bidirectional relationship) [16]. An inverse relationship between adulthood BMI and AMP (mean age: 49.8 years, standard deviation (SD): 5.1) was found in an MR study using the UK Biobank, with replication in the ReproGen consortium [17]. However, there have been limited MR studies investigating potential bidirectional causal relationships between adiposity and reproductive factors other than with AAM and AMP.

We aimed to estimate the causal effects of childhood and adulthood adiposity, independently of each other, on a range of women’s reproductive factors. Additionally, we aimed to investigate the potential effects of reproductive factors on adulthood adiposity, independently of other reproductive factors, as we have shown reproductive factors to be genetically correlated with each other [18]. The findings from this study may highlight the impact that having a healthy weight at different ages can have on women’s menstrual and reproductive function.

## Methods

### UK Biobank

The UK Biobank study is a large population-based cohort of 502,682 individuals who were recruited at ages 37–73 years across the UK between 2006 and 2010. The study includes extensive health and lifestyle questionnaire data, physical measures, and biological samples from which genetic data has been generated. The study protocol is available online, and more details have been published elsewhere [19]. At recruitment, the participants gave informed consent to participate and be followed up.

### Reproductive factors

The reproductive factors investigated in this study were: AAM, AMP, age at first live birth, age at last live birth, number of live births, age first had sexual intercourse (AFS), lifetime number of sexual partners (at the time of assessment) and parous status (ever/never given birth at the time of assessment). Age at first live birth, age at last live birth and number of live births will hereafter be referred to as age at first birth, age at last birth (ALB) and number of births, respectively. In the UK Biobank, these reproductive factors were derived from questionnaire responses at the baseline assessment; further details can be found in Additional file 1.

### Adiposity measures

We assessed adiposity in childhood using the comparative body size measure obtained from the baseline questionnaire in the UK Biobank. Participants were asked, ‘When you were 10 years old, compared to average would you describe yourself as:’ and were given the options: ‘Thinner’, ‘Plumper’ and ‘About average’. In addition, we investigated adiposity in adulthood using body size based on BMI. BMI was derived from height and weight measured during the initial UK Biobank Assessment Centre visit. The categorical body size measure was composed on three groups based on the same proportions as the childhood body size measure [8].

The genetic score of childhood body size has been validated as a marker of childhood adiposity by previous studies including the Trøndelag Health Study [20], The Young Finn Study [21], and the Avon Longitudinal Study of Parents and Children [8], and the polygenic score for childhood body size in the UK Biobank was more correlated with childhood obesity in an independent sample compared to the adulthood BMI genome-wide association studies (GWAS) [22]. In addition, genetic risk scores for childhood body size are more strongly associated with fat mass compared to lean mass, and therefore a suitable measure to use for childhood adiposity [23].

### GWAS

To identify genetic variants robustly related to each of the reproductive factors, we performed GWAS for each reproductive factor among women in the UK Biobank. Each GWAS was performed using the Medical Research Council Integrative Epidemiology Unit UK Biobank GWAS pipeline. Further details on this pipeline [24, 25], and the quality control of genetic data implemented have been described elsewhere [26]. BOLT-LMM was used to conduct the analysis in the GWAS pipeline [27], which accounts for population stratification and relatedness using linear mixed modelling. Genotyping chip and age were included as covariates. Genome-wide significant SNPs were selected at *p* < 5 × 10^−8^ and were clumped to ensure independence at linkage disequilibrium (LD) *r*^2^ < 0.001 and a distance of 10,000 kb using the TwoSampleMR package [25].

We obtained female-only GWAS summary statistics for childhood and adulthood body size from Richardson et al. (2020) [8], where they performed GWAS using a similar approach (Additional file 1) [8].

### Univariable Mendelian randomization

We conducted MR analysis using the ‘TwoSampleMR’ R package [25]. The inverse variance weighted (IVW) method was used in the primary analysis to assess the causal relationships.

First, we assessed the causal effect of childhood body size on the eight reproductive factors. We then investigated the effects of adulthood body size on seven of these reproductive factors, excluding AAM which precedes adulthood. Finally, we assessed the effect of each of the eight reproductive factors on adulthood body size. Since all of the reproductive events occur after childhood, the effect of these on childhood body size was not considered.

The series of univariable MR (UVMR) analysis performed are shown in Additional file 2: Table S1. GWAS estimates were standardized (mean = 0 and SD = 1) prior to performing MR.

Further details can be found in Additional file 1 [28].

### Evaluating the impact of sample overlap, winner’s curse and weak instruments

We used MRlap, a method which is robust to bias introduced by sample overlap, winner’s curse and weak instruments [29]. This method only works in a univariable setting. MRlap was performed using the UK Biobank GWAS summary statistics for reproductive factors and adiposity measures, consistent with our primary univariable analysis.

### Evaluating univariable Mendelian randomization assumptions

To evaluate the strength of genetic instruments, we calculated the F statistic and used a threshold of 10 for determining whether an instrument was sufficiently strong [30]. To evaluate whether the genetic instruments are pleiotropic, we performed MR using additional methods: Weighted mode [31], Weighted median [32], and MR Egger [33, 34]. We also applied MR-PRESSO (Mendelian Randomisation Pleiotropy RESidual Sum and Outlier) to identify and correct for potential outliers (*p* < 0.05) [35]. We used additional methods to evaluate heterogeneity, pleiotropy and intended causal direction. Further details can be found in Additional file 1 [30–38].

### Multivariable Mendelian randomization

We performed multivariable MR (MVMR) analyses, an extension of MR [39, 40], to estimate the direct effects of each reproductive factor and adiposity measure by accounting for the genetic correlation between reproductive factors, and between adiposity measures in childhood and adulthood. Further details can be found in Additional file 1 [39, 40].

These analyses used the ‘MVMR’ R package [41], to estimate the direct effects of childhood and adulthood body size, mutually adjusting for the other adiposity measurement. This mutual adjustment aimed to account for genetic correlation between childhood and adulthood body size.

Finally, in the analyses evaluating the direct effect of each of the eight reproductive factors of interest on adulthood body size, we adjusted for other reproductive factors in turn. The reproductive factors adjusted for were chosen as they have previously been found to have a causal relationship with the reproductive trait under investigation in each MVMR model [18]. The exposure, outcome and adjustment variables included in each MVMR model are shown in Additional file 2: Table S2.

### Evaluating multivariable Mendelian randomization assumptions

We evaluated the joint instrument strength for the two exposures in the MVMR setting using the Sanderson–Windmeijer conditional F statistic [42]. To evaluate evidence of horizontal pleiotropy we used a modified form of Cochran’s Q statistic [41]. Where we identify weak instruments, using a threshold of an F statistic below 10, and/or evidence of pleiotropy, we additionally performed MVMR with minimized Q statistic allowing for weak instruments and heterogeneity [41]. However, we did not use this method where the F statistic is below 4 as the method does not perform well. Further details can be found in Additional file 1 [9, 38, 41, 42].

### Replication analyses

In the primary analysis, we performed two sample MR methods solely in the UK Biobank and therefore the exposure and outcome samples fully overlap, which can lead to bias affecting resulting estimates [43]. Our analysis is also susceptible to a potential winner’s curse, which is the overestimation of the SNP effects on the exposure in a discovery GWAS [44, 45]. Further details can be found in Additional file 1 [43–46]. Given these concerns, we performed replication analyses using samples independent of UK Biobank to evaluate the robustness of our results in both the UVMR and MVMR models.

We obtained GWAS summary statistics from the Early Growth Genetics (EGG) consortium for childhood BMI (39,618 children) [47], GIANT consortium for adulthood BMI (171,970 women) [48], ReproGen consortium for AAM (182,416 women) [49], and AMP (69,360 women) [50], and Social Science Genetic Association Consortium (SSGAC) for AFB (189,656 women) and number of births (225,230 women) [51]. All replication GWAS summary statistics were female-only other than those from the EGG consortium which were sex-combined.

For each relationship assessed in the primary analysis, we firstly used the replication consortia as the exposure and UK Biobank as the outcome, and secondly, vice versa where UK Biobank as the exposure and replication consortia as the outcome. There were no replication GWAS summary statistics available for AFS, ALB, ever parous status, or lifetime number of sexual partners, therefore some relationships could not be replicated as described above. Further details on the proportion of sample overlap and data sources can be found in the Additional file 2: Table S3.

## Results

### UK Biobank

Two hundred and seventy-three thousand, two hundred and thirty-eight women from the UK Biobank were included. The mean age at assessment was 56 years (SD = 8), further sample characteristics are shown in Table 1.

**Table 1:** UK Biobank study characteristics

**UKBB trait**		***N***	**Mean (SD)**
Age at menarche (years)		243,898	13.0 (1.6)
Age first had sexual intercourse (years)		219,486	19.1 (3.6)
Age at first live birth (years)		203,606	25.9 (5.1)
Age at last live birth (years)		203,356	30.1 (5.2)
Age at menopause (years)		143,791	49.7 (5.1)
Number of live births		250,746	1.8 (1.2)
Adulthood body mass index		250,746	27.1 (5.2)
**UKBB trait**		***N***	**Median (IQR)**
Lifetime number of sexual partners (at time of assessment)		208,274	3 (4)
**UKBB trait**		**% (*N*)**	
Never parous (at time of assessment)		18.69 (49,358)	
Childhood body size	About average	50.47 (135,399)	
	Thinner	31.80 (85,316)	
	Plumper	17.74 (47,585)	

*N* sample size, *SD* standard deviation, *IQR* interquartile range

### UK Biobank GWAS

Table 2 displays the number of SNPs associated with each reproductive factor and adiposity measure at genome-wide significance (*p* value < 5 × 10^−8^) after LD clumping within the full UK Biobank sample. In the univariable analysis, all traits had an F statistic over the standard threshold of 10 (Table 2). However, in the multivariable analysis, the conditional F statistic was reduced for all traits (Additional file 2: Table S1) and was below 10 for AFS, AFB, ALB, number of births, ever parous status, and lifetime number of sexual partners with adjustment for other reproductive factors in the MVMR analysis (Additional file 2: Table S4). There were 7 SNPs that overlapped between the genetic instruments for childhood and adulthood body size, 1 between AMP and AFB, and 3 between AFB and ALB in the UK Biobank (Additional file 2: Table S4).

**Table 2:** Instrument strength of each trait of interest.* N* sample size, *nSNPs* number of SNPs at genome-wide significance (p < 5 x 10^-8^) after LD clumping

Trait	*N*	nSNPs	*R*^2^	F statistic
Age at menarche	243,898	223	6.42 × 10^−2^	74.95
Age first had sexual intercourse	219,486	53	9.44 × 10^−3^	39.46
Age at first live birth	203,606	41	8.38 × 10^−3^	41.97
Age at last live birth	203,356	9	1.92 × 10^−3^	43.46
Number of live births	250,746	9	1.68 × 10^−3^	46.75
Ever parous status	250,746	4	8.59 × 10^−4^	53.92
Age at menopause	143,791	84	4.74 × 10^−2^	85.08
Lifetime number of sexual partners	208,274	34	6.36 × 10^−3^	39.20
Childhood body size	246,511	112	3.74 × 10^−2^	85.48
Adulthood body size	246,511	160	3.23 × 10^−2^	51.39

### Mendelian randomization

#### Effects of childhood body size on reproductive factors

**Fig. 1:**
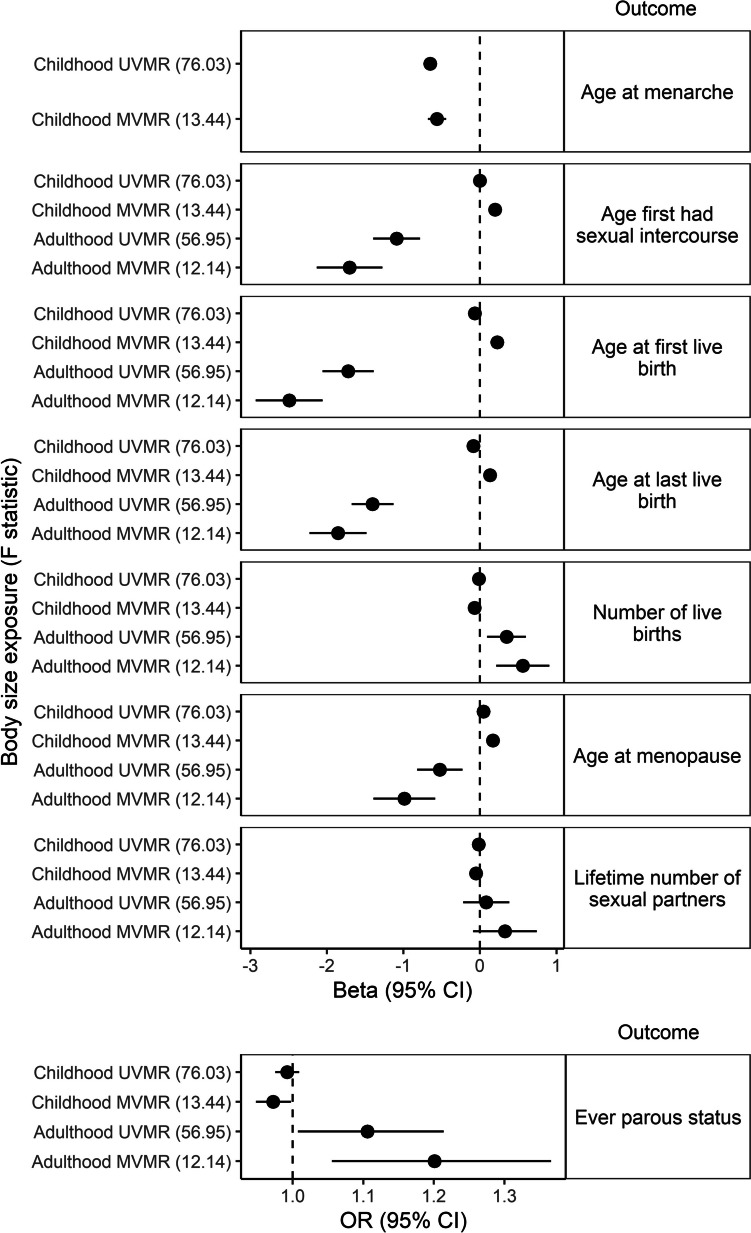
Univariable Mendelian randomization (UVMR) and multivariable Mendelian randomization (MVMR) findings: the effects of childhood body size (MVMR adjusted for adulthood body size) and adulthood body size (MVMR adjusted for childhood body size) on reproductive factors in the UK Biobank. GWAS regression coefficients were standardized prior to performing MR. *CI* Confidence interval

Findings referred to here are shown in Fig. 1 and in Additional file 2: Table S5–S6. All effects are displayed as per 1 SD increase in the exposure.

We found an inverse effect of childhood body size on AAM in both the UVMR (beta (*B*) =  − 0.65 SD, 95% confidence interval (CI) =  − 0.74, − 0.57) and MVMR analysis, which adjusts for adulthood body size (*B* =  − 0.56 SD, CI =  − 0.68, − 0.44).

The UVMR analysis revealed inverse effects of childhood body size on AFB (*B* =  − 0.07 SD, CI =  − 0.12, − 0.01), ALB (*B* =  − 0.09 SD, CI =  − 0.13, − 0.04), and no evidence for an effect on AFS (*B* = 0.00 SD, CI =  − 0.06, 0.05) or AMP (*B* = 0.05 SD, CI =  − 0.01, 0.11). Conversely in the MVMR model, adjusting for adulthood body size, the effects were positive for AFS (*B* = 0.20 SD, CI = 0.11, 0.28), AFB (*B* = 0.22 SD, CI = 0.14, 0.31), ALB (*B* = 0.13 SD, CI = 0.06, 0.21) and AMP (*B* = 0.17 SD, CI = 0.09, 0.25).

Findings from the UVMR analysis suggested there is no evidence for an effect of childhood body size on the number of births (*B* =  − 0.01 SD, CI =  − 0.06, 0.04), or ever parous status (odds ratio (OR) = 0.99, CI = 0.98, 1.01). However, in the MVMR model, adjusting for adulthood body size, there was evidence for inverse effects on the number of births (*B* =  − 0.07 SD, CI =  − 0.14, − 1.74 × 10^−3^) and ever parous status (OR = 0.97, CI = 0.95, 1.00).

There was no evidence for an effect of childhood body size on the lifetime number of sexual partners in the UVMR model (*B* =  − 0.02 SD, CI =  − 0.08, 0.04) or in the MVMR model, adjusting for adulthood body size (*B* =  − 0.05 SD, CI =  − 0.13, 0.03).

#### Effect of adulthood body size on reproductive factors

Findings referred to here are shown in Fig. 1 and in Additional file 2: Table S5–S6. All effects are displayed as per 1 SD increase.

In the UVMR model we identified inverse effects of adulthood body size on AFS (*B* =  − 1.09 SD, CI =  − 1.40, − 0.78), AFB (*B* =  − 1.72 SD, CI =  − 2.06, − 1.39), ALB (*B* =  − 1.40 SD, CI =  − 1.68, − 1.13), and AMP (*B* =  − 0.53 SD, CI =  − 0.82, − 0.23). These effects were maintained or strengthened in the MVMR analysis adjusting for childhood body size; AFS (*B* =  − 1.70 SD, CI =  − 2.13, − 1.28), AFB (*B* =  − 2.48 SD, CI =  − 2.93, − 2.06), ALB (*B* =  − 1.86 SD, CI =  − 2.23, − 1.48) and AMP (*B* =  − 0.99 SD, CI =  − 1.39, − 0.59).

The UVMR analysis revealed evidence for a positive effect of adulthood body size on the number of births (*B* = 0.35 SD, CI = 0.10, 0.60) and ever parous status (OR = 1.11, CI = 1.01,1.21) which were maintained in the MVMR model adjusting for childhood body size (*B* = 0.56 SD, CI = 0.21, 0.91 and OR = 1.20, CI = 1.06, 1.37).

There was no evidence for an effect of adulthood body size on the lifetime number of sexual partners in both the UVMR model (*B* = 0.08 SD, CI =  − 0.22, 0.38) and MVMR model after adjusting for childhood body size (*B* = 0.33 SD, CI =  − 0.09, 0.74).

#### Effect of reproductive factors on adulthood body size

**Fig. 2:**
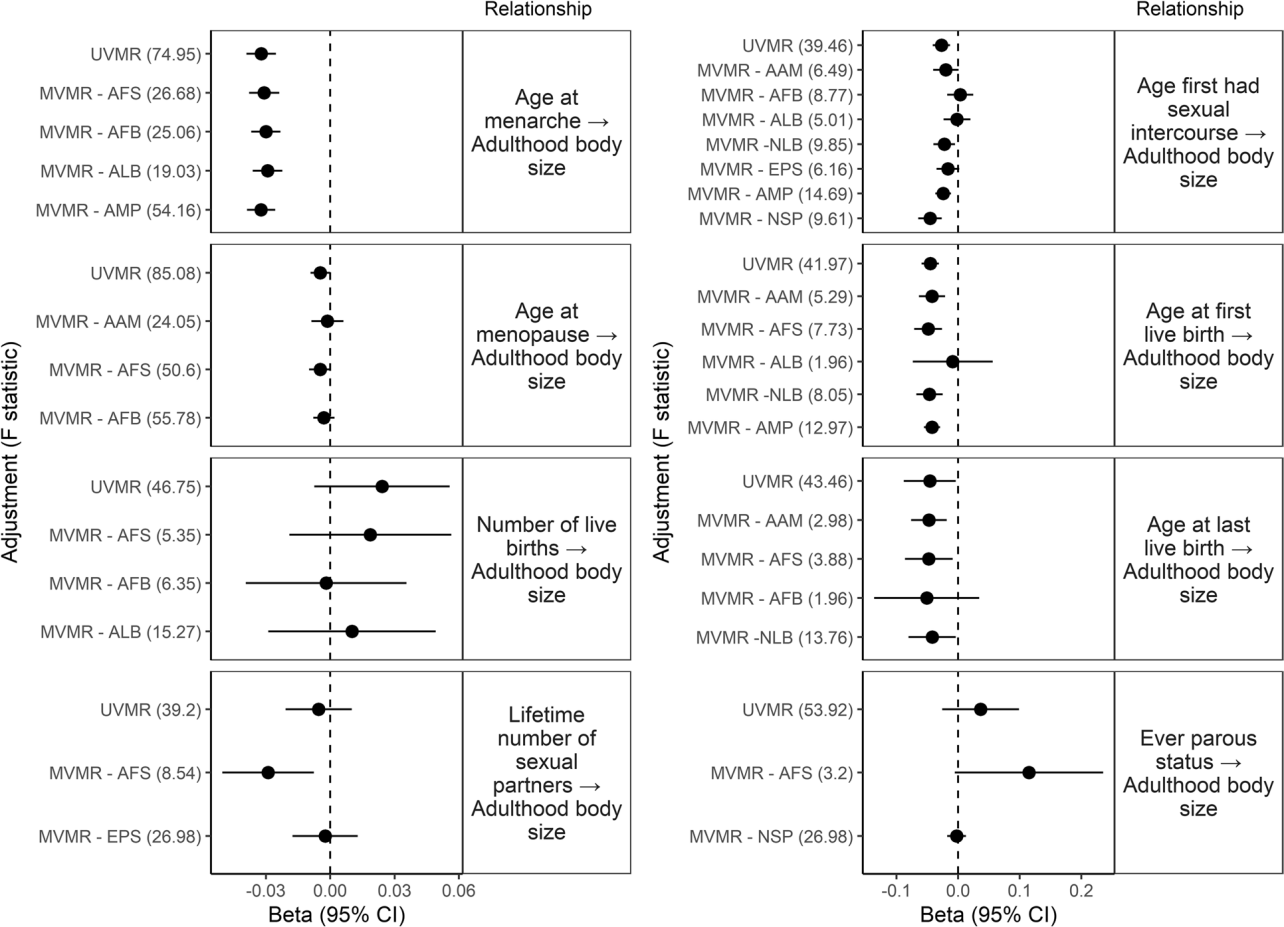
Univariable Mendelian randomization (UVMR) and multivariable Mendelian randomization (MVMR) findings: the effects of reproductive factors on adulthood body size in the UK Biobank. GWAS regression coefficients were standardized prior to performing MR. *AAM* Age at menarche, *AFS* Age first had sexual intercourse, *AFB A*ge at first live birth, *ALB* Age at last live birth, *NLB* Number of live births, *EPS* Ever parous status, *AMP* Age at menopause, *NSP* Lifetime number of sexual partners, *CI* Confidence interval

Findings are displayed in Fig. 2 and in Additional file 2: Table S5–6. All effects are displayed as per 1 SD increase.

The UVMR analysis of AAM on adulthood body size revealed evidence for a small inverse effect (*B* =  − 3.22 × 10^−2^ SD, CI =  − 3.90 × 10^−2^, − 2.53 × 10^−2^) which did not change with adjustment for relevant reproductive factors in the MVMR. We also found small inverse effects of AFS (*B* =  − 2.70 × 10^−2^ SD, CI =  − 4.12 × 10^−2^, − 1.29 × 10^−2^), AFB (*B* =  − 4.52 × 10^−2^ SD, CI =  − 5.91 × 10^−2^, − 3.13 × 10^−2^), ALB (*B* =  − 4.59 × 10^−2^ SD, CI =  − 8.80 × 10^−2^, − 3.90 × 10^−3^) and AMP (*B* =  − 4.62 × 10^−3^ SD, CI =  − 9.22 × 10^−3^, − 2.59 × 10^−5^) on adulthood body size. However, these effects completely attenuated after adjustment for the relevant reproductive factors in the MVMR models. While we did not find evidence for an effect of the lifetime number of sexual partners in the UVMR model (*B* =  − 5 × 10^−3^, CI =  − 0.02, 0.01), adjustment for AFS revealed a small inverse effect (*B* =  − 0.03 SD, CI =  − 0.05, − 7.64 × 10^−3^).

### Evaluating Mendelian randomization assumptions

Details on findings of analyses evaluating MR assumptions can be found in Additional file 1 and Additional file 2: Table S7–15.

#### Univariable MR

We found that the IVW method was consistent with the additional MR methods; MR Egger, weighted median and weighted mode, for the effects of childhood body size on AAM, and AAM on adulthood body size. The IVW method was inconsistent with at least one of these additional methods for the other 12 effects identified in the UVMR (Additional file 2: Table S9). While there were some inconsistencies between the effects identified using these additional MR methods and the IVW method (Additional file 2: Table S9), suggesting evidence of pleiotropy, the MR PRESSO method revealed little change in the strength of evidence after adjustment for outliers (Additional file 2: Table S11).

MRlap findings suggest bias may have arisen due to sample overlap, and that the effect of adulthood body size on reproductive factors may be overestimated by ~ 19% (range: 15–28%) while the effect of each reproductive factor on adulthood body size may be underestimated by ~ 12% (range: 8–17%). Estimates corrected for possible bias due to sample overlap using MRlap are shown in additional file 2: Table S12.

#### Multivariable MR

For the most part, instrument strength in MVMR is greatly reduced compared to the UVMR analysis. Since there was evidence of heterogeneity across all relationships (Additional file 2: Table S13), MVMR with minimized Q statistic was performed which corrects for heterogeneity and weak instruments. However, in the analysis of AFB adjusted for ALB, ALB adjusted for AAM, AFS and AFB, and ever parous status adjusted for AFS, the F statistic was below 4 so we could not use this method. Where it could be performed, this approach revealed similar strength of evidence across most MVMR analyses (Additional file 2: Table S14). Of note the effect of AMP on adulthood body size (adjusting for AFS) revealed evidence for a very small inverse effect (*B* =  − 4.07 × 10^−3^ SD, CI =  − 6.74 × 10^−3^, − 5.78 × 10^−4^ per 1 SD increase), which was not revealed in the primary multivariable analysis.

### Replication analyses

We performed the UVMR and MVMR analysis using additional non-UK Biobank GWAS summary statistics where possible. Table 3 and Additional file 2: Table S16 display the number of SNPs associated with each reproductive factor and adiposity measure, at genome-wide significance (*p* < 5 × 10^−8^) after LD clumping, for the UVMR and MVMR analyses respectively.

**Table 3:** Instrument strength of each replication trait of interest. *N* sample size, *nSNPs* number of SNPs at genome-wide significance (*p* < 5 × 10^−8^) after LD clumping

Trait	*N*	nSNPs	*R*^2^	F statistic
Childhood body mass index (from EGG)	39,618	16	0.0236	59.89
Adulthood body mass index (from GIANT)	171,970	37	0.0145	68.25
Age at menarche (from ReproGen)	182,416	60	0.0261	81.52
Age at menopause (from ReproGen)	69,360	39	0.0420	77.89
Age at first birth (from SSGAC)	189,656	5	9.14 × 10^−4^	34.70

Of the effects identified in the primary UVMR analysis, we replicated the effect of childhood body size on AAM and the effects of adulthood body size on AFB, ALB, number of births and AMP. In addition, we replicated the effect of AAM, AFS, and AFB on adulthood body size (Additional file 2: Table S17).

Of the effects identified in the primary MVMR analysis, we replicated the effect of childhood body size on AAM adjusted for adulthood body size, and the effects of adulthood body size on AFB and number of births adjusted for childhood body size. Finally, we replicated the effects of AAM (adjusted for AFS, AFB, ALB and AMP), AFS (adjusted for AMP and lifetime number of sexual partners) and AFB (adjusted for AAM, number of births and AMP) on adulthood body size (Additional file 2: Table S18).

It is worth noting that the UVMR model of adulthood body size on the number of births and AMP, (Additional file 2: Table S17), and MVMR model of childhood body size on the number of births (adjusted for adulthood body size) and adulthood body size on AFB and number of births (adjusted for childhood body size) (Additional file 2: Table S18), were only replicated where the replication GWAS summary statistics were used for the outcome.

## Discussion

In this study, we used both UVMR and MVMR to investigate the causal relationships between childhood and adulthood adiposity measures and reproductive factors.

We show evidence that earlier AAM leads to a higher adulthood body size independent of other reproductive factors. In addition, after accounting for the genetic correlation between childhood and adulthood body size, we identified opposing direct effects of childhood and adulthood body size on AFS, AFB, ALB, AMP and the likelihood of having children, suggesting adiposity in earlier life may affect reproductive factors through different mechanisms compared to later in life. Furthermore, we found evidence that higher adulthood body size leads to a higher number of children born.

It is worth highlighting that the aim with using MVMR methods in this study was to assess whether childhood body size has an impact on reproductive outcomes independently of adulthood body size, for example through permanent effects on organs and systems rather than effects driven by tracking of adiposity across the lifecourse. This could demonstrate whether or not the effects of childhood body size are potentially reversible if people normalize their weight trajectory later in life. For some reproductive outcomes, we observed a change in the direction of effect, i.e. the total effect of childhood body size on age at first birth and age at last birth is negative in UVMR models, but the direct effect in MVMR models is positive. It is important to point out that the direct effect of childhood body size from a MVMR model represents the effect of a one-unit higher childhood body size, holding adulthood body size constant. In contrast, the total effect from a UVMR model represents the effect of a one-unit higher body size in childhood, which on average, will equate to a higher body size in adulthood. Therefore, it is not unreasonable for these two comparisons to lead to opposing directions of effect since it is plausible that adiposity experienced at different times in the lifecourse may influence reproductive traits differently.

Using additional genetic consortia, we replicated the direct effects of AAM on adulthood body size, childhood body size on AAM, and adulthood body size on AFB and number of births. However, it is worth noting that where effects were not replicated, this may be a consequence of weak genetic instruments.

Weak instruments in the replication MVMR analysis were a particular concern for analyses of the effects of reproductive factors on adulthood body size, adjusting for other relevant reproductive factors. For example, previous work has revealed that AFB and ALB are highly genetically correlated (*r*_*g*_ = 0.94, *p* < 0.001) and causally linked (IVW *B* = 0.72, CI = 0.67, 0.77) [18]. Therefore, in the MVMR the instruments for both factors are weak when adjusting for the other, making it difficult to isolate the direct effects of AFB and ALB.

In analyses of childhood and adulthood body size on AMP, genetic instruments were strong, but the relationships were not replicated. This suggests overestimation of effects in the primary MVMR analysis may be due to bias arising from the winner’s curse [44, 45], or sample overlap between the exposure and outcome GWAS populations [43]. We additionally used the MRlap method to correct for bias arising from sample overlap in the UVMR analysis. We identified the same directionality of effect compared to the primary analysis; however, the effect of adulthood body size on reproductive factors was slightly overestimated, while the effects of each reproductive factor on adulthood body size were slightly underestimated in the primary analysis.

We identified a direct inverse effect of adulthood body size on AFS. Given that the first experience of sexual intercourse commonly occurs prior to adulthood (in the UK Biobank, 64% of women had sexual intercourse before the age of 20), we assume that the genetic variants identified in relation to adulthood body size in the UK Biobank, where participants were assessed between the ages of 40 and 70 years, are stable across adulthood and are therefore valid instruments for early adulthood adiposity. A similar assumption was made in a recent study which assessed the effects of childhood and adulthood adiposity on smoking initiation (mean age 17.8 years) [9]. In support of this, an evaluation of the HUNT study identified the crossover of variance explained by childhood to adulthood adiposity genetic scores occurs in late adolescence/early adulthood [20], suggesting that the genetic liability of adiposity from childhood to adulthood changes during puberty and is stable thereafter. Nevertheless, it may be useful to consider a third adiposity measure in relation to AFS, using a GWAS performed in late adolescence or early adulthood which, to the best of our knowledge, is not currently available.

It may seem biologically implausible to adjust for adulthood body size in the MVMR analysis between childhood body size and AAM due to the temporal order of these factors. However, the MVMR analyses aim to account for the underlying genetic correlation with adulthood adiposity, hence why adulthood body size was included in the MVMR model [9].

### Potential mechanisms

Our findings show evidence that higher childhood body size leads to an earlier AAM which supports findings from previous observational and MR studies [2, 16]. This is likely due to increased production of adipocytokines from adipose tissue which may influence pubertal timing [52], and additionally due to increased adiposity leading to increased leptin levels which is necessary for the onset of puberty [53].

It has been suggested that higher adulthood BMI leads to a later AMP due to the effects of adipose tissue leading to increased oestrogen and other endogenous hormone levels [54]. While we found a higher childhood body size leads to a later AMP, we found that higher adulthood body size reduced AMP, with an inverse relationship also being observed in replication analyses, although of a smaller magnitude. This may be due to higher BMI depleting ovarian reserves, which causes menopause to occur earlier [6].

Our findings suggest earlier AAM leads to higher adulthood body size, which concurs with previous research [1, 14, 15]. This is likely due to menarche leading to increased exposure to endogenous hormones such as oestrogen and progesterone which causes physical changes to occur earlier [55].

We found evidence that higher adulthood body size leads to a higher number of births and having children. Previous work has suggested that increased adiposity and obesity contribute to subfertility due to insulin resistance and increased activity of adipocytes, which in turn may lead to reduced endocrine responses in women such as decreased production of oestrogens and luteinizing hormone, and a greater androgen production [56]. Furthermore, there is evidence that increased BMI can lead to conditions associated with decreased fertility [57]. Previous research suggests low adiposity leads to subfertility due to undernutrition. This may occur as a lower level of hormones such as leptin are produced by adipose tissue which have a role in reproductive functioning [58, 59].

Previous research has shown a J-shaped association between BMI and subfertility [60]; however, it is worth noting that our analysis only evaluated linear effects. Nevertheless, later AFB in the UK Biobank may not be related to reduced fertility but rather reflect a choice to have children later in life. This is consistent with our findings of a higher body size in adulthood leading to an earlier AFB. It is worth considering that this differs from the positive effect we identified of childhood body size on AFB. This suggests there may be opposing biological and social mechanisms in action depending on when higher adiposity is experienced across the lifecourse.

Finally, higher adiposity in childhood, in comparison, delayed AFS. This may be explained by being overweight in adolescence resulting in later engagement in sexual activity due to social stigma [61–63].

### Limitations

This study had a number of limitations. Firstly, data on the reproductive factors investigated as well as early life body size have been derived from self-report. AMP is likely to be captured reasonably accurately in the UK Biobank because the time between menopause and reporting AMP will be relatively short due to the age of the cohort (40–70 years of age). Nevertheless, the reliability of reporting is likely to decline with the amount of time passed since menopause [64]. We can be more confident that reports of AFB, ALB and number of births are accurate since these are significant life events that are likely to be reliably recalled. However, given the long time between the experience and the report, AAM may not be as reliably recalled [65]. Furthermore, self-report of AFS and lifetime number of sexual partners may not be accurately estimated [66]. While adulthood body size was captured from measurements collected during the initial UK Biobank Assessment Centre visit, childhood body size was derived from questionnaire data so may be subject to recall bias and could capture perceived body size. However, as we highlight in the methods section, the genetic score of this measure has been validated as a marker of childhood adiposity by previous studies [8, 20, 21]. Similarly, there has typically been good replication of the genetic scores for the reproductive traits in other cohorts [49–51].

The childhood BMI GWAS from the EGG consortium used in the replication analyses was not sex-specific and had a lower sample size compared to the measure from the UK Biobank. The lower sample size reduced instrument strength, and childhood BMI in boys was additionally captured which may have influenced the results. Unfortunately, there was not a sex-specific childhood adiposity GWAS available to use for replication analysis.

Finally, we were not able to use MVMR with the minimized Q statistic, which aims to obtain estimates which are robust to weak instruments and pleiotropy, for MVMR analysis with fewer than four genetic instruments. This is because this statistic does not perform well with this number of variants and consequently is not reliable. Therefore, further work would be required to untangle the effects of AFB, ALB and ever parous status on adulthood body size. In addition, AFB, AFS, number of births, and ever having children, are bio-social traits. Therefore, findings are not easily generalisable to settings with different social norms in the UK and to more contemporary populations. We found evidence for the effects of childhood body size and adulthood body size on AFB and ALB, although sensitivity analysis demonstrated difficulty separating out the effects of AFB and ALB, likely due to a very high genetic correlation between these traits.

## Conclusions

In summary, we found evidence for direct effects of childhood body size on age at menarche, and of age at menarche on adulthood body size. We additionally identified some evidence for the direct effects of childhood and adulthood body size on a number of other reproductive factors. Of note, the effects of childhood and adulthood body size had opposing effects on numerous reproductive factors, including age first had sexual intercourse, age at first birth, age at last birth, number of births, ever parous status, and age at menopause.

This study demonstrates the importance of considering a lifecourse approach when investigating the inter-relationships between adiposity measures and reproductive events, as well as the use of ‘age specific’ genetic instruments when evaluating lifecourse hypotheses in a Mendelian randomization framework. Furthermore, our findings have implications in guiding future studies interested in investigating the causal effects of adiposity on women’s health and understanding mechanisms which may lead to menstrual and reproductive disorders. Finally, this work highlights the impact that having a healthy weight at different ages can have on women’s menstrual and reproductive function.

### Supplementary Information

**Additional file 1.** Further details.

**Additional file 2: Table S1.** Relationships explored in the univariable analysis. **Table S2.** Relationships explored in the multivariable analysis with adjustments. **Table S3.** Replication consortia and information. **Table S4.** Multivariable mendelian randomization instrument strength and SNP overlap. **Table S5.** Univariable mendelian randomization findings – IVW method. **Table S6.** Multivariable mendelian randomization findings - IVW method. **Table S7.** Steiger filtering. **Table S8.** Univariable mendelian randomization analyses – heterogeneity test. **Table S9.** Univariable mendelian randomization analyses - additional MR methods. **Table S10.** Univariable mendelian randomization analyses – Egger intercept test. **Table S11.** Univariable mendelian randomization analyses- MR PRESSO. **Table S12.** Univariable mendelian randomization analyses- MRlap. **Table S13.** Multivariable mendelian randomization analyses – heterogeneity test. **Table S14.** Multivariable mendelian randomization with minimised Q statistic findings. **Table S15.** Multivariable mendelian randomization analysis of childhood body size on reproductive factors - steiger filtered SNPs removed. **Table S16.** Replication multivariable mendelian randomization instrument strength. **Table S17.** Replication univariable mendelian randomization findings - IVW method. **Table S18.** Replication multivariable mendelian randomization findings - IVW method.

## Data Availability

The availability of all data analysed in this study has been referenced throughout the manuscript and supplementary materials. GWAS summary statistics for age at menarche and menopause from the ReproGen consortium: https://www.reprogen.org/. GWAS summary statistics for age at first birth and number of births from the SSGAC consortium: https://www.thessgac.org/. GWAS summary statistics for childhood BMI from the EGG consortium: http://egg-consortium.org/. GWAS summary statistics for adulthood BMI from the GIANT consortium: https://portals.broadinstitute.org/collaboration/giant/index.php/Main_Page.

## Abbreviations

AAM: Age at menarche
AFB: Age at first birth
AFS: Age first had sexual intercourse
ALB: Age at last birth
AMP: Age at menopause
BMI: Body mass index
EGG: Early Growth Genetics
GIANT: Genetic Investigation of Anthropometric Traits
GWAS: Genome-wide association studies
LD: Linkage disequilibrium
MR: Mendelian randomization
MVMR: Multivariable Mendelian randomization
SD: Standard deviation
SSGAC: Social Science Genetic Association Consortium
UVMR: Univariable Mendelian randomization
