# Supplementary material for: Establishing the relationships between adiposity and reproductive factors: a multivariable Mendelian randomization analysis
Source: BMC Med. 2023 Sep 12;21:350. doi: 10.1186/s12916-023-03051-x (PMC10496263; doi:10.1186/s12916-023-03051-x)
Supplement: Supplementary file 1 — Additional file 1. Further details. [file 12916_2023_3051_MOESM1_ESM.docx]

**Methods**

## UK biobank: Reproductive factors

Age at menarche was derived from the question: "How old were you when your periods started?". Age at menopause was derived from the question "How old were you when your periods stopped?", this did not include women who had a hysterectomy/were not sure whether they had gone through menopause. To identify the number of live births, women were asked "How many children have you given birth to? (Please include live births only)". A binary measure of parous status, coded as “0” or “1”, where women who had or had not given birth, was derived from the measure of number of live births. Women who indicated that they had given birth to one child were asked "How old were you when you had your child?". Women who indicated that they had given birth to more than one child were asked "How old were you when you had your FIRST child?" and "How old were you when you had your LAST child?". To derive age at first live birth and age at last live birth, responses from primiparous and multiparous women were combined. Women were asked, "What was your age when you first had sexual intercourse? (Sexual intercourse includes vaginal, oral or anal intercourse)" and "About how many sexual partners have you had in your lifetime?", collected from all participants except those who indicated they never had had sexual intercourse. Responses to these questions were used to derive age first had sexual intercourse and lifetime number of sexual partners, individuals who had not had sexual intercourse were coded as “0” in the number of sexual partners analysis. We performed rank-based inverse normal transformation on lifetime number of sexual partners given the skewed distribution of this variable, all other reproductive factors were untransformed.

## GWAS

We obtained GWAS summary statistics for childhood and adulthood body size from Richardson et al. 2020,[8] here they performed a GWAS similarly to the reproductive factors using BOLT-LMM accounting for population stratification and relatedness and including age at baseline and genotyping array as covariates.

## Univariable mendelian randomization

The IVW MR method combines Wald ratios, calculated by dividing the SNP-outcome association by the SNP-exposure association, in a multiplicative random effect meta-analysis where the weight of each ratio is the inverse of the variance of the SNP-outcome association.[28]

This method makes a number of assumptions: that the genetic instruments are strongly associated with the exposure; do not share common causes with the outcome; and are not pleiotropic i.e., do not have an effect on the outcome through a pathway other than via the exposure.[28]

## Evaluating univariable mendelian randomization assumptions

To evaluate the strength of the genetic instruments, we determined the mean F statistic for each trait, which is calculated based on the variance explained (r^2^) by the genetic instrument and sample size of the exposure.[30]

In addition, we assessed evidence for heterogeneity and invalid instruments, which can imply presence of pleiotropy.[36,37] We evaluated this calculating the Cochran's Q statistic between instruments using the TwoSampleMR package.

To evaluate whether the genetic instruments are pleiotropic we performed MR using additional methods: Weighted mode,[31] Weighted median,[32] and MR Egger.[33, 34] The intercept and 95% confidence interval of the MR-Egger regression line was used to determine directional pleiotropy using the TwoSampleMR package.[33]

We also applied the R function MR-PRESSO (Mendelian Randomisation Pleiotropy RESidual Sum and Outlier) to identify and correct for potential outliers (p <0.05).[35]

We performed the MR Steiger test and Steiger filtering bi-directionally for the relationships between reproductive factors and adulthood body size where we found evidence for bidirectional relationships.[38] This was performed to assess whether the hypothesized causal directional of the relationship was correct for each genetic instrument.[38]

## Multivariable mendelian randomization

Multivariable MR (MVMR) is an extension of MR, and the assumptions are also extended. These assumptions are that the genetic instruments must be strongly associated with each exposure given the other exposures included in the model; do not share any common cause with the outcome; and are independent of the outcome given all of the exposures.[39,40]

## Evaluating multivariable Mendelian randomization assumptions

We evaluated the joint instrument strength for the two exposures in the MVMR setting using the Sanderson–Windmeijer conditional F-statistic,[42] this was calculated using the ‘strength_mvmr()’ function from the “MVMR” R package.[41]

To evaluate evidence of horizontal pleiotropy we used a modified form of Cochran’s Q statistic available from the ‘pleiotropy_mvmr()’ function from the “MVMR” R package.[41]

Where we identify weak instruments and/or evidence of pleiotropy we additionally performed MVMR with minimized Q-statistic allowing for heterogeneity, using the ‘qhet_mvmr()’ function from the “MVMR” R package.[41]

Conditioning on adulthood body size in the assessment of the effect of childhood body size on reproductive factors that may have an effect on adulthood body size can cause collider bias.[9] To avoid this collider bias we first performed the MR Steiger test between adulthood body size and each reproductive factor to identify SNPs that explain more variation on the reproductive factor than adulthood body size.[38] We then performed the MVMR analysis investigating the effects of childhood body size on reproductive adjusting for adulthood body size excluding those Steiger test identified SNPs.

## Replication analyses

In the primary analysis, we performed two sample MR methods solely in UK Biobank and therefore the exposure and outcome samples fully overlap. Large overlap in the sample(s) used to generate genetic variant-exposure and genetic variant-outcome associations can introduce bias in estimates obtained using two-sample MR methods, which could lead to an overestimation of effects.[43] However, it has been suggested that applying two-sample MR methods in a single sample may be performed within large studies with minimal bias.[46] Since the GWAS used to identify genetic instruments for MR analysis were also identified in UK Biobank, our analysis is also susceptible to potential winner’s curse, which is the overestimation of the SNP effects on the exposure in a discovery GWAS.[44, 45]

Given these concerns, we performed replication analyses using samples independent of UK Biobank to evaluate the robustness of our results. Further details on the number of studies and sample sizes used for the replication consortia are shown in **additional file 2: Table. S3**.

**Results**

## Steiger filtering for bidirectional relationships

We applied the MR Steiger method to each univariable MR (UVMR) model of reproductive factors and adulthood body size, where we found evidence of an effect, to assess whether we had captured the intended causal direction. We evaluated this for the relationship between age first had sexual intercourse, age at first birth, age at last birth and age at menopause in relation to adulthood body size. Findings show aggregated instruments have successfully captured the intended causal direction in all cases (**Additional file 2: Table. S7**).

## Evaluating UVMR assumptions

We identified evidence for heterogeneity in the individual SNP effects in the IVW across all UVMR models with the exception of the relationship between ever parous status and adulthood body size (**Additional file 2: Table. S8**). We therefore investigated the robustness of results using additional MR methods to account for potential pleiotropy, MR Egger, Weighted median, and Weighted mode. For most relationships, results using these methods were inconsistent with the IVW method, with the exception for the effects of childhood body size on age at menarche, and age at menarche on adulthood body size, which were consistent across these methods. Results from these methods can be found in **additional file 2: Table. S9**.

### MR-Egger intercept test

For the primary UVMR models, the MR-Egger intercept test revealed evidence for directional pleiotropy in the relationship between childhood body size and age at menarche, adulthood body size and age first had sexual intercourse, age at first birth, age at last birth, age at menopause and ever parous status (**Additional file 2: Table. S10).**

### MR-PRESSO

MR-PRESSO revealed outlier SNPs in all the primary UVMR models, which were likely to be driving the levels of heterogeneity. However, after outlier correction, there was little change in the strength of evidence apart from for models of childhood body size on age at menopause where an inverse effect emerged, and models of number of births and adulthood body size where evidence for a positive effect emerged with outlier correction (**Additional file 2: Table. S11**).

## MRlap

MRlap findings show a reduction in the magnitude of effects when assessing the effect of adulthood body size on reproductive factors. In addition, we show an elevation in the magnitude of effect when assessing the effect of each reproductive factor on adulthood body size (**Additional file 2: Table. S12**). In both directions the evidence of effects that were identified in the primary UVMR models, were maintained. This suggests bias has arisen due to sample overlap that acts in the opposite direction depending on which direction we are investigating, i.e., effect of adulthood body size on reproductive factors, or reproductive factor to adulthood body size.

## Evaluating MVMR assumptions

For all relationships assessed in the MVMR primary analysis, we identified evidence of heterogeneity in the individual SNP in the IVW across all investigated relationships (**Additional file 2: Table. S13**).

Due to evidence of heterogeneity, we additionally performed MVMR with minimised Q-statistic allowing for heterogeneity. These analyses revealed similar strength of evidence across analyses other than for the relationships between childhood body size and ever parous status (adjusting for adulthood body size), adulthood body size and number of births (adjusting for childhood body size), adulthood body size and ever parous status (adjusting for childhood body size), and age at last birth and adulthood body size (adjusting for number of births), where effects attenuated. Of note the effect of age at menopause on adulthood body size (adjusting for age first had sexual intercourse) revealed evidence for a very small inverse effect (B=-4.07x10^-3^ SD, CI=- 6.74 x10^-3^, -5.78 x10^-4^ per 1 SD increase) (**Additional file 2: Table. S14**).

However, this method does not perform well where the instrument strength is less than or equal to 5. We therefore we did not investigated effects of age at first birth on adulthood body size (adjusting for age at last birth), age at last birth on adulthood body size (adjusting for age at menarche, age first had sexual intercourse, and age at first birth) and ever parous status on adulthood body size (age first had sexual intercourse).

We removed Steiger test identified SNPs from the MVMR analysis assessing the effects of childhood body size on reproductive factors adjusting for adulthood body size, however there were no Steiger identified SNPs between adulthood body size and ever parous status. There was no change in evidence compared to the primary MVMR analysis other than the effect of childhood body size on number of births attenuated slightly, with 95% confidence intervals crossing the null. In addition, instrument strength remained similar to the primary MVMR analysis. (**Additional file 2: Table. S15**)

## Replication analyses

In the univariable analysis all replication traits had a F statistic over the standard threshold of 10, however, in the multivariable analysis the F statistic was reduced to ~3.8 for both childhood and adulthood adiposity, and to 0.9 for age at first birth (from SSGAC) when adjusted for number of births (from SSGAC) (**Additional file 2: Table. S16**).
